# Consequences of Delayed Diagnosis in Treatment of Retinoblastoma

**Published:** 2014-07-30

**Authors:** Mohammad Faranoush, Amir Abbas Hedayati Asl, Azim Mehrvar, Narjes Mehrvar, Rokhsaneh Zangooei, Ehsan Abadi, Mardawig Alebouyeh, Maryam Tashvighi

**Affiliations:** 1 MAHAK Pediatric Cancer Treatment and Research Center; 2 Iran University of Medical Sciences; 3 Army Medical Sciences University, Tehran; 4 Islamic Azad University of Medical Sciences (Qom Branch), Qom, Iran

**Keywords:** Childhood Malignancy, Eye Tumor, Delayed Diagnosis, Retinoblastoma

## Abstract

***Objective:*** One of the primary factors in managing patients with retinoblastoma is early diagnosis. The main idea of this study was to recognize the consequences of delay in diagnosis on therapy of the disease.

***Methods:*** A retrospective review of all children with proven retinoblastoma, who had presented to MAHAK hospital in Tehran, from April 2007 to Dec 2011, was performed. Grouping of intraocular tumors was applied as A to E according to International Classification of Retinoblastoma.

***Findings*** ***:*** There were 157 (91 boys) children eligible for study. The mean age was 1.21±0.11 years with average delay in diagnosis of 3.4±0.53 months. Classification of D group in both unilateral (93 patients) and bilateral tumors was the largest category. A significant relation (*P*=0.05) between delayed diagnosis time and tumor grouping was evident. The most frequent symptoms were leukocoria and strabismus. Age was significantly lower in the subgroup of bilateral tumors than in unilateral retinoblastomas (0.6±0.12 year vs 1.6±0.15 years). The diagnosis was delayed in subgroup of extra ocular retinoblastoma more than in intraocular tumors (8.7±2.9 months vs 2.9±0.52 months).

***Conclusion:*** The authors recommend early referring of suspected cases to ophthalmologists and pediatric oncologists and to organize educational programs to publisize signs and symptoms of the disease such as leukocoria, strabismus and ocular inflammatory disorders through national media. In conclusion, early diagnosis of retinoblastoma can be the primary factor in managing the patients as the delay in diagnosis accounts for highly advanced disease and poor prognosis.

## Introduction

Retinoblastoma, a malignant intraocular tumor, is commonly detected by parents seeing general signs of this disease^[^^1^^,^^2^^]^. In spite of being a rare childhood cancer, it is a common primary ocular tumor in children^[^^3^^,^^4^^]^. the incidence rate of retinoblastoma is approximately 1 in 15000 and 1 in 16600 live births in the United States and northern Europe respectively ^[^^5^^,^^6^^]^. This malignancy is more frequent in Latin America, Africa and India^[^^7^^,^^8^^]^. 

 During past 80 years, the curability rate has been dramatically increased to more than 90%^[^^9^^,^^10^^]^. The five years overall survival rate in retinoblastoma is greater than 90%^[^^11^^-^^13^^]^. Early diagnosis shortly after the detection of symptoms can improve survival rate^[^^14^^]^. The delay in clinical diagnosis depends on the medical culture of the country and awareness of the parents^[^^14^^]^. The estimated time interval between the first symptoms and clinical diagnosis can be a landmark of risk factor for altering survival rate in retinoblastoma, as the late diagnosis results in poor prognosis^[^^14^^,^^15^^]^. 

 In order to investigate the consequences of delayed diagnosis in treatment of children with retinoblastoma, we conducted this study in MAHAK Pediatric Cancer Treatment and Research Center (MPCTRC), a referral childhood malignancy center in Tehran, Iran.

## Subjects and Methods

We undertook a retrospective review of all children with retinoblastoma, who were admitted to MPCTRC from April 2007 to Dec 2011. Only patients with clinically confirmed tumor by pediatric ophthalmologist were included in the study. Information for each patient including sex, age at diagnosis, the clinical manifestations, date of first symptoms, date of diagnosis, date of initiation of therapy, laterality and stage of the tumor was recorded. 

 MPCTRC, being a large and well-known referral childhood malignancy center in the capital, receives children with retinoblastoma referred for chemotherapy from the whole country.

 Based on the clinical examinations and according to International Classification of Retinoblastoma (ICRB), groupings were applied as A to E for intraocular retinoblastoma^[^^16^^,^^17^^]^. 

 Data was analyzed by SPSS version 19. Comparison of mean values was performed using the non-parametric Mann-Whitney test for unpaired samples, and chi-squared for parametric variables. All *P*-values were two-sided and considered statistically significant when <0.05.

## Findings

The inclusion criteria were met by 157 patients (n=91, 58% boys). The mean age was 1.21±0.11 years (median 1.0 years; interquartile range 0-2 years; range: 0 to 6 years). The mean interval between the first detection of symptoms and the clinical diagnosis was 3.4±0.53 months (median 1 month; interquartile range 5.7–88.7 days; range 0-36.5 months). A delay in the diagnosis of less than 5 months was found for 105 (66.9%) children, and 9 (5.7%) patients were diagnosed with retinoblastoma longer than 15 months after the first symptoms were recognized. 

 In 93 (59.2%) children, the tumor was unilateral. Intraocular tumor was found in 141 (89.8%) patients. Classification of D in children with bilateral tumor formed the largest group (n=40 eyes; 38.5%), followed by E (n=27 eyes; 26%), B (n=16 eyes; 15.4%), C (n=12 eyes; 11.5%) and finally by the A group (n=9 eyes; 8.6%). Children with unilateral tumor showed class D as the largest group (n=37 eyes; 45.1%), followed by E (n=28 eyes; 34.1%), C (n=9 eyes; 11.1%), B (n=7 eyes; 8.5%) and finally by the A group (n=1 eye; 1.2%). Chi-squared test showed the significant relation (*P*=0.05) between tumor grouping and delayed diagnosis in enrolled patients (median delay time: group A 1.2±0.7 months; group B 0.2±0.1 months; group C 1.5±0.6 months; group D 3.6±0.9 months; group E: 4.8 ±1.06 months). 

**Fig. 1:**
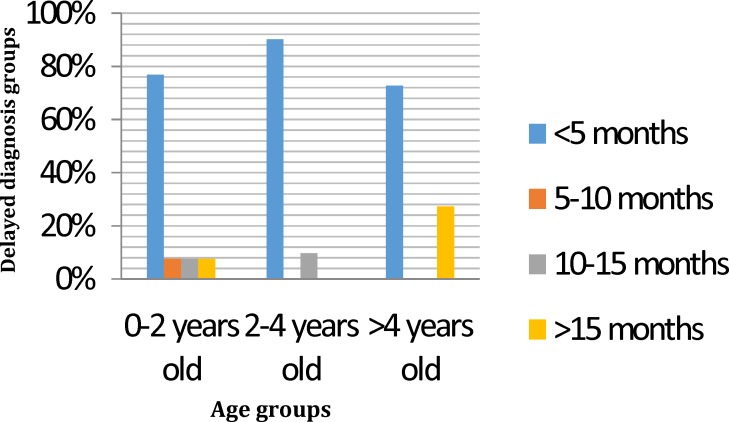
Age groups and delayed diagnosis

 Categorizing the whole enrolled patients by age into subgroups of 0-2 years, 2-4 years and more than 4 years showed that the diagnostic delay was significantly (*P*<0.05) the longest in the elder aged group of more than 4 year olds (7.6±3.8 months; median 11.5 days; interquartile range 4.5 days to 18.7 months; range 0-36.5 months) compared with the youngest age group 0-2 years (3.5±0.6 months; median 1.0 month; interquartile range 6.5 to 94 days; range 0-29.3 months ) and the middle-aged group 2-4 years (2.1±0.53 months; median 1.0 month; interquartile range 5 to 65 days; range 0-14.13 months) (Fig. 1). 

**Fig. 2:**
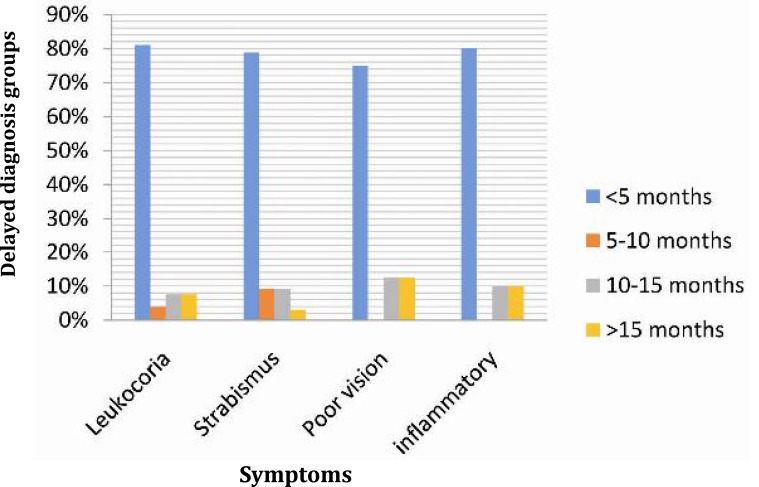
Presenting symptoms of retinoblastoma

 The most frequent symptoms were leukocoria in 106 (67.51%) children with a mean delay in the diagnosis of 3.4±0.58 months (median 1 month; interquartile range 7 to 88 days; range 0-29.3 months) (Fig. 2). Strabismus was the leading symptom for 33 (25.4%) children, in whom the mean diagnostic delay was 2.9±0.7 months (median 31 days; interquartile range 10 to 121 days; range 0-18 months). As shown in Fig 2, presence of poor vision and eye inflammation predicted a late diagnosis of the tumor.

The most frequent symptoms were leukocoria in 106 (67.51%) children with a mean delay in the diagnosis of 3.4±0.58 months (median 1 month; interquartile range 7 to 88 days; range 0-29.3 months) (Fig. 2). Strabismus was the leading symptom for 33 (25.4%) children, in whom the mean diagnostic delay was 2.9±0.7 months (median 31 days; interquartile range 10 to 121 days; range 0-18 months). As shown in Fig 2, presence of poor vision and eye inflammation predicted a late diagnosis of the tumor.

**Fig. 3:**
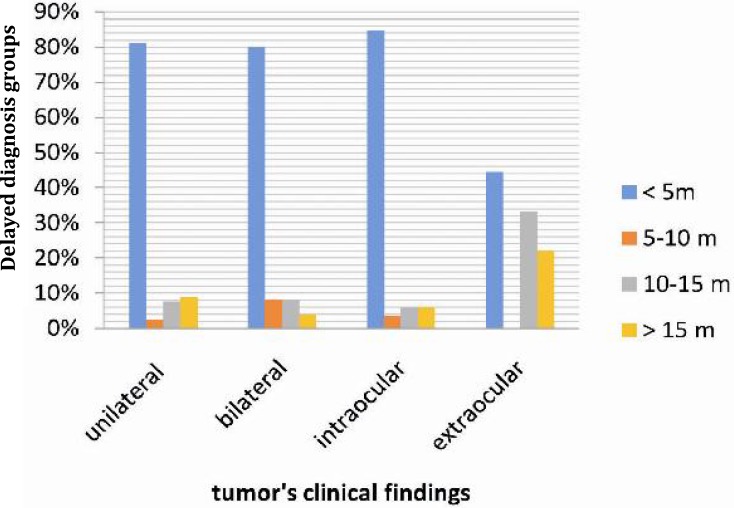
The clinical patterns of retinblastoma

 Taking the whole study population, Fig. 3 shows the association of the length of diagnostic delay and clinical finding of the tumor. It can be seen that extra ocular location of bilateral retinoblastoma was found in children with a diagnostic delay of 10 to 15 months, while extra ocular location with unilateral retinoblastoma was distinguished with a diagnostic delay of more than 15 months. 

 Stratifying the whole study population into patients with unilateral retinoblastoma (n=93, 59.2%) and patients with bilateral retinoblastoma (n=64, 40.8%) revealed that age was significantly (*P*=o.oo1) lower in the subgroup with bilateral retinoblastomas than in the subgroup with unilateral retinoblastomas (0.6±0.12 years; median 0.1 year; interquartile range 0-1 year; range 0-5 years vs 1.6±0.15 years; median 1.5 years; interquartile range 0-3 years; range 0-6 years).

The delay in the diagnosis of the tumor did not vary significantly between the subgroup with unilateral retinoblastomas (mean 3.9±0.87 months; median 1 month; interquartile range 7.2-91.7 days; range 0-36.5 months), and the subgroup with bilateral retinoblastoma (mean 2.6±0.62 months; median 27days; interquartile range 1.7-61 days; range 0-24.3 months). 

 The delay in the diagnosis of the tumor varied significantly (*P*=0.006) between the subgroup with intraocular retinoblastomas (mean 2.9±0.52 months; median 1 month; interquartile range 6-61 days; range 0-36.5 months), and the subgroup with extra ocular retinoblastoma (mean 8.7±2.9 months; median 3 months; interquartile range 5 days-14.2 months; range 0-24.3 months).

 Between 2007 and 2011, retinoblastoma was one of the most frequent solid tumors admitted to MPCTRC, accounting for 9.6% of pediatric malignancies. Retinoblastoma represents 6.1% of all cancers in children <5 years of age in the United States^[^^6^^]^. In our study, the mean age of patients was 1.21±0.11 years (59.2% unilateral), was consistent with the literature. Approximately 80% of patients are diagnosed when <4 years old^[^^14^^]^; 60% of cases represent as unilateral and the remaining bilateral^[^^1^^,^^2^^]^. 

 Retrospective analysis of 157 cases of retinoblastoma between age and laterality pattern at the time of diagnosis revealed that bilateral retinoblastoma presents at an earlier age than unilateral retinoblastoma. This finding supports the Knudson "two-hit" hypothesis of retino-blastoma genetics^[^^16^^]^. 

 The key point in the early diagnosis of retinoblastoma is referring to pediatricians who can detect ocular disorders, which parents do not perceive most times. Distinguishing the signs and symptoms, funduscopic screening and initiation of therapy as soon as possible are of paramount importance^[^^14^^]^. 

 Ophthalmic disorders can present as strabismus and leukocoria^[^^14^^]^. Studies conducted in USA, UK, Brazil and China state that leukocoria is the most frequent symptom^[^^3^^,^^14^^]^. This was found in 67.51% of cases in our series, followed by strabismus (25.4%) and eye inflammation (7%). Literature reviews demonstrated that 50-60% of cases were diagnosed by leukocoria, 25% by strabismus and 6-10% by inflammation^[^^14^^]^. Bai and coworkers found the most frequent symptoms were leukocoria and poor vision, in addition to presence of strabismus related to late diagnosis of the tumor^[^^3^^]^. 

 The mean delay in the diagnosis of retinoblastomas in Iranian children treated in the MPCTRC was 3.4±0.53 months. Duration of symptoms for longer than 10 months in enrolled patients was a prognostic factor for poor outcome. Rodriguez-Gallindo by reviewing cases of retinoblastoma at Brazil in 1995 demonstrated that 68% of patients showed a delay in diagnosis shorter than six months^[^^7^^]^. Bai and Rodrigues by different screening showed that delay diagnosis in their cases was 4.1 and 8.3 months respectively in China (2011) and Brazil (2004)^ [^^3^^,^^14^^]^. In our study, the risk of delayed diagnosis was the same in both groups of patients with strabismus (2.9±0.7 months) and leukocoria (3.4±0.58 months), while Rodrigues et al found that cases with strabismus showed higher risk of delayed diagnosis (8.8 months) than cases with leukocoria (5.6 months)^ [^^14^^]^. 

 The diagnostic delay was significantly longer in the age group > 4 years old than other age groups (*P*<0.05). While Bai et al showed this relation in the age group of 2-4 years. 

 There was a predominance of patients with intraocular tumor, which shows shorter duration of symptoms compared to those with advanced extra ocular tumors. Rodrigues et al reported improvement of prognosis in patients with intraocular retinoblastomas^[^^14^^]^. This improvement can be due to focusing on early recognition of retinoblastoma by public awareness. In our study, the patients with extra-ocular tumor exhibited signs for over 10 months, and both parents and ophthalmologists failed to make an early diagnosis. Patients with low educational level living in the rural and countryside showed advanced disease. Thus, the influence of educational awareness on parents and health services can lead to overcome delayed diagnoses.

## Conclusion

According to these facts, for an infant/child with positive family history of retinoblastoma, early referring to ophthalmologist and pediatric oncologist is recommended. In addition, educational programs should be offered to bring into public awareness symptoms of retinoblastoma such as leukocoria, strabismus and eye inflammation to result in early diagnosis. Early diagnosis of retinoblastoma can be the critical risk factor in managing the patients as delay in diagnosis accounts for highly advanced tumor and poor prognosis.
